# Minocycline-induced DRESS: could dosage in relation to body fat mass be a contributing factor?

**DOI:** 10.3389/fphar.2025.1681474

**Published:** 2025-09-25

**Authors:** Vincent Descamps

**Affiliations:** Department of Dermatology, Bichat Hospital, APHP, Paris Cité University, Paris, France

**Keywords:** adverse drug reaction, DRESS (drug reaction with eosinophilia and systemic symptoms), minocyclin, weight adjustable, risk factor

## Perspective

I read with great interest the excellent review on minocycline-associated DRESS (Drug Reaction with Eosinophilia and Systemic Symptoms) recently published by Pan and Wu in *Frontiers in Pharmacology* (Pan and Wu, 2025).

DRESS is classified as a type IV hypersensitivity reaction. Interestingly, for certain drugs, the risk of developing this severe cutaneous adverse reaction appears to be linked to the initial dosage at the start of treatment (Blumenthal et al., 2025). This is well documented for antiepileptic drugs, allopurinol, and sulfasalazine. For these medications, a gradual dose escalation is recommended when initiating therapy, which significantly reduces the incidence of DRESS.

It would be of particular interest to have data on the initial dosing of minocycline in reported cases of DRESS. Demographic data from existing reports indicate that most cases involve young women, whose average body weight may be lower than the general population. However, it is not standard clinical practice to adjust minocycline dosing according to body weight, and doses of 100 mg/day or even 200 mg/day are frequently prescribed.

Furthermore, minocycline is a lipophilic drug that tends to accumulate in adipose tissue—a characteristic that could potentially increase exposure and risk in women.

It would therefore be valuable to analyze the dosage-to-body weight ratio in minocycline-associated DRESS cases, especially in female patients. Such data could inform future guidelines and support the adoption of lower starting doses—similar to current recommendations for many antiepileptic drugs and allopurinol—to minimize the risk of DRESS.
